# Restoration of a Nonvital Tooth with Fiber Reinforce Composite (Wallpapering Technique)

**DOI:** 10.1155/2020/9619787

**Published:** 2020-06-05

**Authors:** Sara Valizadeh, Ladan Ranjbar Omrani, Simone Deliperi, Farzaneh Sadeghi Mahounak

**Affiliations:** ^1^Restorative Dentistry Department, School of Dentistry, Tehran University of Medical Sciences, Tehran, Iran; ^2^Tufts University School of Dental Medicine, Boston, MA, USA; ^3^Private Practice, Cagliari, Italy; ^4^Restorative Dentistry Department, School of Dentistry, Shahid beheshti University of Medical Sciences, Tehran, Iran

## Abstract

**Introduction:**

Reconstruction of endodontically treated tooth (ETT) is one of the greatest challenges in dentistry. Clinical success of fiber reinforcement composite (FRC) restorations in ETT depends on many factors like remaining tooth structure, knowing advantages of adhesive dentistry besides its drawbacks, and the correct use of fibers in combination with resin composite. *Case Report*. This article presents a case in which fibers have been used in composite buildup in order to increase the toughness and strength of the ETT direct restoration. In addition, this technique does not require root canal enlargement to eliminate the risk of root perforation. Also, this one visit treatment can be helpful for patients that could not pay the cost of indirect restoration and/or have no time.

**Conclusion:**

It seems in selected patients with special considerations, FRC composite restoration is valid alternatives for indirect restoration.

## 1. Introduction

Without placing the coronal restoration, root canal treatments were not considered complete. Restoration of nonvital teeth is always challenging for dentists.

Appropriate treatment plan selection should be based on remaining tooth structure, cavity wall thickness, tooth position in the arch, and load applied to the tooth [1].

Previously, endodontically treated teeth (ETT) were reconstructed automatically with post and core and full crown. But this treatment plan had many risks like root perforation, sacrificing a considerable amount of sound tooth structure, and tooth fracture [2].

By modern adhesive dentistry, some alternative methods are proposed for ETT restoration in accordance with minimally invasive dentistry. Composite improvements in regard of physical and mechanical properties, besides esthetic appearance, were led to the progressive use of these dental materials and more tooth tissue preservation. This was ideal for patients who could not afford the cost of the indirect restorations [3].

However, the most important drawback of resin composites, which is polymerization shrinkage that produces stresses in the interface of tooth and restoration, has still been existed [4].

There are several methods for compensating these stresses due to the composite shrinkage.

One of them is an incremental technique for composite insertion in the cavity. The other way for controlling stress is light-curing techniques like soft start or ramp mode [5].

Clinicians should keep in mind that composite resin restoration is a rigid material; it lacks toughness but does not lack of strength or stiffness.

Material resistance to rapid propagation of cracks called toughness and it is an inherent material property, which can be used for predicting structural performance [6].

Fiber-reinforced composite (FRC) restoration has been introduced to increase durability in composite restoration, enhance composite stiffness, and provide better force distribution along fibers. FRC development has increased the use of composite resin materials in extensive preparations [7].

Due to the mode of failure of composite and weakened walls in ETT, it is advised to insert fibers against the cavity walls to avoid failure. New techniques and materials help the practitioner to solve old problems from a different perspective and achieve innovative and unique solutions [8].

This article describes a clinical approach in the reconstruction of ETT posterior teeth in two cases by direct fiber-reinforced composite (FRC) restoration, as well as critical discussion of the advantages and disadvantages.

## 2. Case Report

**Figure 1:**
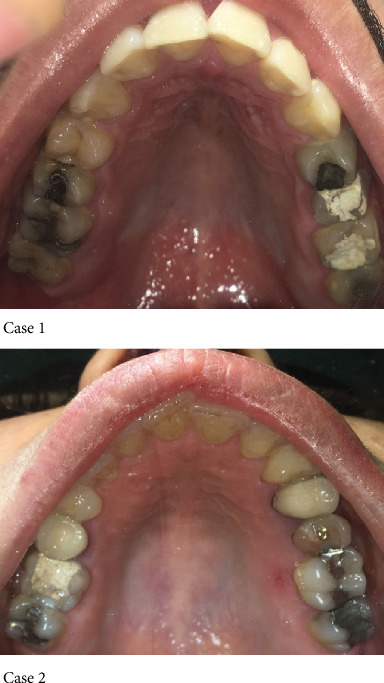
Preoperative view of tooth #16 and #26 with temporary restoration after root canal therapy.

**Figure 2:**
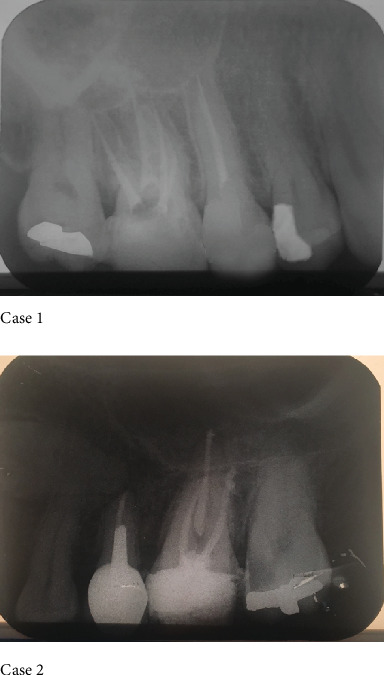
Preapical radiographic of #16 and #26 teeth after root canal therapy.

A 23-year-old male patient presented with an endodontically treated first right upper molar with two weeks delay after completing endodontic therapy. The second case was a 35-year-old female that had root canal therapy on the upper left first molar after 3 weeks delay after RCT (Figure 1). Preapical radiographies of the upper first molar of these two cases after root canal therapy are seen in Figure 2.

Because the whole cavosurface margins were on the enamel, it was explained to the patient that the treatment plan of choice was the placement of an indirect ceramic onlay. Alternatively, because of the intact mesial marginal ridge, a direct cuspal coverage restoration with resin composite could be an option. After discussing the treatment plan with the patient for both direct and indirect restorations and getting an informed consent, fiber-reinforced direct composite (FRCDC) restoration was selected for the upper first molars due to the financial issues.

It was decided to follow the six-step stress-reduced direct composite (SRDC) Protocol from Deliperi et al. [8, 9].

The first step for SRDC protocol is “Analysis of the occlusion” to avoid either overloading or lack of centric stops in occlusal area. So preoperative occlusal analysis was done and showed even distribution of the occlusal load on the whole of residual tissue of tooth #16; thus, we decided not to reduce mesiofacial and mesiopalatal cusps.

**Figure 3:**
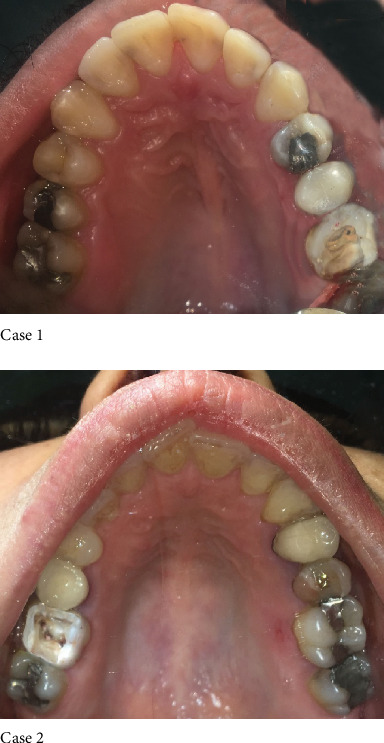
Occlusal view of #16 and #26 after removing temporary restoration.

The second step is “Cavity Preparation and Caries Removal End Points”. Thus existing temporary restoration was removed using #2 and #4 round burs (Tizkavan, Iran). The cavity was prepared in a very conservative manner, being sure to remove all the decayed dental tissue and trying to preserve the remaining sound tooth structure as much as possible according to the basic guidelines for direct minimally invasive dentistry. Residual sharp angles and unsupported enamel were smoothed. No bevels were placed on the occlusal and gingival margins (Figure 3). The main goals of step 2 were to avoid the formation of any sharp line angle on either the prepared enamel or dentin and to preserve the peripheral rim for bonding.

In the third step, “Analysis of Residual Tooth Structure” was done. Once the preparation was complete, it was determined that the distolingual cusp was unsupported. However, the thickness of the residual facial walls was greater than 2 mm, and the preservation of the entire mesial marginal ridge was sufficient to support an FR-SRDC restoration.

For the fourth step, we “Prepare the Dental Substrate to Achieve a Reliable Bond to Enamel and Dentin”. A Tofflemire Matrix Retainer and band were placed around teeth #16 and #26, using a wooden wedge and burnishing the metal matrix provided good adaptation to the gingival margin. Isolation was provided by means of cotton roll and suction. The tooth was etched for 15 seconds using a 37% phosphoric acid (Meta etchant, Bio Med, Korea). The etchant was removed, and the cavity was rinsed with water for 30 seconds, being careful to maintain a moist surface. A two-step etch and rinse ethanol-based adhesive system (adper single bond 2, 3M ESPE, USA) was placed in the preparation and gently air thinned and light cured for 20 seconds using an LED curing light (woodi pecker, China).

**Figure 4:**
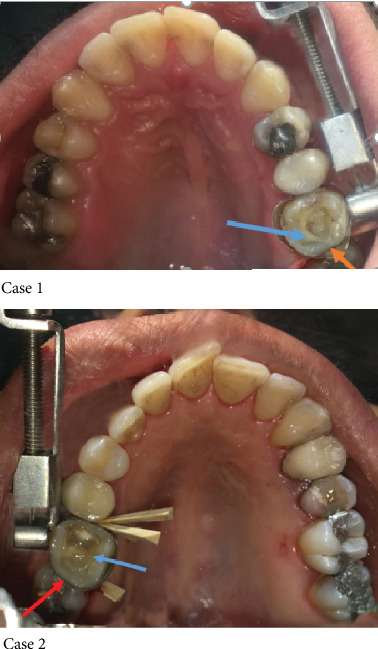
Peripheral enamel skeleton was built up in the distal part (Red Arrow) using wedge-shaped increments and placing appropriate length fiber in close contact with a palatal cusp (Blue Arrow).

For “Control of Polymerization Stresses” in the fifth step, the missing peripheral tooth structure was reconstructed with the 2 mm wedge-shaped composite increments. Filtek Z250 (3M ESPE, USA) microhybrid composite resin was used to restore the distal marginal ridge (Red Arrow in Figure 4). Filtek Z250 A3 shade was used to build up the cervical third of both the distopalatal and distal surfaces of the tooth. The rest of the distal surface and distopalatal wall was completed using the Filtek Z250 A2 shade in order to make two-third of the reconstruction lighter than the gingival part. Then, matrix retainer, band, and wedges are removed.

1. The sixth step followed by “Wallpapering Dentin Walls with Polyethylene Fiber Strands” was done by the selecting of the correct length and width of the fibers. For measuring the accurate length of the fiber, a dental probe (Hu-Friedy, Chicago, IL, USA) was used to measure the mesiodistal distance of cavity walls. Two preimpregnated glass fiber (Interlik, Angelus, Brazil) strand pieces (4 mm wide and 11 mm length) were cut. Fibers can be cut with a microtome and low-speed cutting machine but, in this case, they were cut with bistoury and no. 11 blade. The cutting edge of the blade was placed in a perpendicular direction to the fibers and with the almost heavy pressure without very much back and forward movements. Fibers were covered with a very thin layer of a flowable composite (Filtek Bulk Fill, 3M ESPE, USA); prior to insertion into the cavity, they were precontoured c-shaped and then carried into the cavity by cotton plier, and fitted into the cavity walls properly with thin composite spatule, bonded, and cured for 20 seconds. (Blue Arrow in Figure 4). They were placed one after the other as described; if two fibers were placed simultaneously, the adaptation to the cavity wall and exact position may be compromised. At the proximal surfaces, the fiber strands overlapped each other. They were placed to the buccal and palatal wall and their extension to the proximal surface overlapped each other approximately 1 to 1.5 mm
2. The key point of reducing polymerization shrinkage stress on the residual weakened walls is decreasing the composite volume between the fibers and tooth structure which is gained by tight adaptation of fibers to the tooth structure. 2 fibers were used in this case because it was decided to splint buccal and palatal cusps together. There are some techniques in the literature using fiber strands inside the composite body, but we choose just 2 fibers for cross splinting

**Figure 5:**
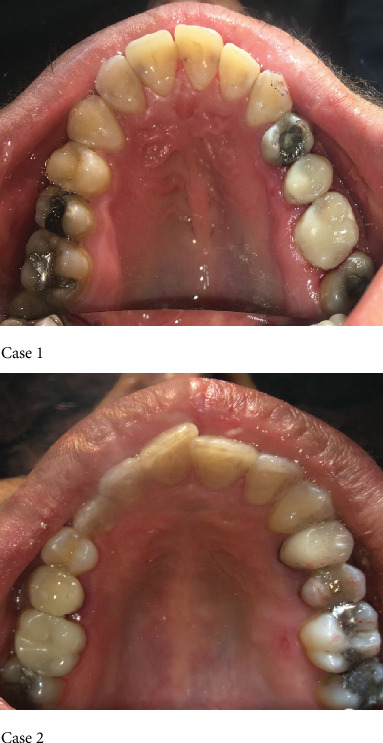
Composite buildup of #16 and #26 teeth.

After that restoration process, “Dentin and Occlusal Surface Buildup” was done. Due to the stress absorbing effect of the fiber strands, a 2 mm thick dentin wedge-shaped increments layers of composite resin was placed into contact with the fiber strand in order to decrease the C-factor ratio. Single increments of A2 shade of Filtek Z250 were applied to each cusp separately, achieving the final occlusal morphology (Figure 5). Postcuring done by irradiating the restored tooth through the facial and palatal surface for 30 s each to complete polymerization.

Finishing was done by diamond finishing bur, and polishing was followed by moulets (Shofu dental, Japan).

Finally, “Occlusal Force Equilibration” performed by occlusal adjustment by locating centric stops on the tooth structure and composite resin simultaneously and by the same intensity to prevent excessive forces and also like the adjacent teeth.

## 3. Discussion

One of the dentistry challenges is the coronal restoration of root filled posterior teeth. The ideal technique for restoration for ETT is still a debate. Loss of dentine structure is the main factor that has an effect on the survival rate of pulpless teeth [10]. Indirect restoration is considered the treatment of choice in endodontically treated teeth (ETT) for many years.

Amalgam builds up and post and cores and crown are being replaced by direct composite and glass-fiber posts, in addition to fiber reinforce composite resin and all ceramic crowns are being often chosen because of their superior aesthetic outcome and avoiding problems like root perforation, more loss of sound tooth structure, and invasiveness of such treatments.

This method has advantages like more fracture resistance and less polymerization shrinkage than composite buildup. But in practice, it is not easy to adapt fibers in precise position on cavity walls and also provide complete isolation and also need more chair time.

Amalgam has been used in direct cusp capping of posterior teeth in the past, and studies have been shown an acceptable long-term survival rate for large amalgam restorations [11].

Tooth strengthening is also the purpose of restoration besides tooth repair.

It seems that the reinforcement of residual tooth structure is possible with modern adhesive systems and composite resins [4]. Functional stresses could be better distributed along the interface of bonding. In addition, with the use of bonded restorations, a more sound tooth structure would be preserved and fracture resistance be increased [12]. But several clinical studies are needed to evaluate the bond durability of these restorations over time [9].

Due to the polymerization shrinkage which is the main drawback of direct composite restorations, dentists may encounter many problems in selecting these restorations [13]. However, Stress Reduced Direct Composite (SRDC) technique could be considered as an alternative to indirect restorations [8].

Although resin-based composites (RBCs) were showing loss of anatomical shape and marginal breakdown in the past, they exhibit more wear resistant and better mechanical characteristics now. Current direct composite restoration wear rate is 10 to 15 *μ*m per year [14].

In order to improve the inherent deficiencies of RBCs, the use of fibers in combination with resin composite in restoring ETT that are structurally compromised, consider as a promising treatment [15]. Fiber Reinforced Composite (FRC) restorations would change the dynamics of stresses at the interface of restoration and tooth. Fracture toughness and flexural strength of RBCs increase with fiber insertion. Fiber design makes a network that leads to a rapid crack growth stopping mechanism [16].

In this case, pieces of fibers were used circumferentially in the closest distance of vertical facial and palatal walls of residual tissue of the teeth and the composite buildup. Belli and others showed that fracture strength increased and cusp movement decrease if fibers were placed against the dentinal wall. It was described also that fibers have influenced in lowering C-factor and enhancing microtensile bond strength [17].

The first step in this case composite buildup was making peripheral skeleton in missing distal marginal ridge to transform multisurface to class I cavity. It can help to reach smooth surface restoration without excess in margins [18].

Wedge-shaped increments are essential for compensating stress from polymerization shrinkage. In fact, the goal of multiple wedge-shaped increments technique is decreasing C-factor by trying no more than two bonded cavity walls at a time [8]. The wallpapering technique which uses fibers circumferentially in contact with vertical walls could absorb lateral forces that are created during occlusal vertical loading. Therefore, decrease failures or if failures happen, the damage is not catastrophic and in most cases is reparable. Higher risk for catastrophic failure will exist if the remaining cavity walls become thinner than 2 mm [19].

In a study done by Kuijs and colleagues, it was shown that there is a comparable failure mode in direct and indirect composite resin restorations regarding fracture [20].

Even ceramic restorations have some issues besides being costly and time-consuming than direct composite restorations. Internal fitness and tooth preparation design play an important role in the success of them and also resin cement wear is another concern that occurs in ceramic restorations [21].

Precise evaluation of patients' criteria is mandatory for selecting the wallpapering technique as an acceptable treatment plan. Parafunctional habits and heavy occlusion make this treatment inappropriate [22].

In the following session after one and half year, no recurrent caries, chipping, or fracture was seen. However, more long-term in vitro and clinical studies are necessary for supporting this type of treatment plan.

## 4. Conclusion

One visit treatment, preservation of sound tooth structure and reduced cost, make RFC restoration an acceptable treatment for ETT reconstruction. It seems that in special cases, minimally invasive treatment could be considered as an alternative for indirect restorations.

## Data Availability

Data are available from the corresponding author on request.

## Additional Points

*Clinical Relevance*. Endodontically treated tooth could benefit from proper use of fibers reinforced composite restoration in some special clinical situations. It not only has acceptable performance but also delaying or preventing complex indirect restorations.
